# Influence of Nitrate and Light on Fucoxanthin Content and Key Gene Expression in the Marine Diatom *Thalassiosira rotula*

**DOI:** 10.3390/plants14213344

**Published:** 2025-10-31

**Authors:** Maria Letizia Madeo, Ida Orefice, Michele Ferrari, Teresa Greca, Leonardo Bruno, Giovanna Romano, Radiana Cozza

**Affiliations:** 1 Department of Biology, Ecology and Earth Sciences, University of Calabria, Via Ponte P. Bucci 6B, 87036 Arcavacata di Rende, Cosenza, Italy; michele.ferrari@unical.it (M.F.); leonardo.bruno@unical.it (L.B.); 2 Ecosustainable Marine Biotechnology Department, Stazione Zoologica Anton Dohrn, Via Acton 55, 80133 Napoli, Napoli, Italy; ida.orefice@szn.it (I.O.); giovanna.romano@szn.it (G.R.); 3 Stazione Zoologica Anton Dohrn, C.da Torre Spaccata, 87071 Amendolara, Cosenza, Italy; teresa.greca@szn.it

**Keywords:** fucoxanthin, *Thalassiosira rotula*, nitrate availability, light conditions, gene expression

## Abstract

Fucoxanthin is the predominant carotenoid in diatoms, playing a central role in light harvesting and photoprotection, and is increasingly valued for its potential in pharmaceutical, nutraceutical, and cosmetic applications. In this study, we investigated the influence of high nitrate supplementation, low-light exposure, and combined treatment, on fucoxanthin content and on the expression of key genes involved in its biosynthetic pathway in the marine diatom *Thalassiosira rotula*. Fucoxanthin content was quantified using HPLC-based and spectrophotometric methods. Control culture at the exponential growth phase showed a fucoxanthin content of 4.7 mg g^−1^ DW, reaching 5.2 mg g^−1^ DW under low-light conditions at the late exponential phase. Gene expression analysis revealed condition-dependent modulation of major biosynthetic genes (*PSY*, *PDS*, *ZCIS*, *CRTISO*, *ZEP*, *VDL*, *DDE*). Early biosynthetic genes, *PSY* and *PDS*, were upregulated under low light, whereas *ZCIS* and *CRTISO* responded to high nitrate availability. *ZEP* exhibited treatment-specific induction and *VDL* isoforms showed differential regulation, highlighting distinct xanthophyll cycle gene expression patterns across treatments. These results demonstrate that both light and nitrate availability modulate fucoxanthin content and biosynthetic gene expression in *T. rotula*, providing insights into the regulatory mechanisms underlying carotenoid metabolism in diatoms and proposing *T. rotula* as a potential candidate for fucoxanthin production.

## 1. Introduction

Fucoxanthin (Fx) is a bioactive xanthophyll carotenoid, primarily found in brown algae and certain microalgae. Its unique chemical structure—characterized by allene and epoxide groups—distinguishes it from other plant carotenoids [1]. Functionally, fucoxanthin plays a crucial role in photosynthesis, enhancing light-harvesting efficiency and protecting cells from oxidative stress due to excess light [1]. Beyond its physiological importance in algae, Fx has gained substantial interest for its pharmacological potential, exhibiting antioxidant, anti-inflammatory, antidiabetic, anti-obesity, antimalarial, and anticancer activities [2,3,4,5]. These properties make it an attractive compound for applications in health, cosmetics, and nutrition.

Currently, the primary sources of Fx are brown macroalgae, such as *Laminaria* spp., *Sargassum* spp., *Fucus* spp., *Undaria pinnatifida*, *Nizamuddinia zanardinii* and *Cystoseira indica* [6,7,8]. However, their relatively low fucoxanthin concentrations result in high extraction costs and limited supply. In general, microalgae present a more affordable and advantageous alternative to brown macroalgae for fucoxanthin production [9,10,11]. Diatoms, in particular, are easily cultivated and can produce large biomass quantities within short timeframes. Notably, marine diatoms, such as *Phaeodactylum tricornutum*, can contain fucoxanthin concentrations significantly higher than those found in brown algae, and in some cases reported to be over an order of magnitude greater, making them a promising and commercially viable source [12]. Other microalgae species have demonstrated high Fx content, ranging from 0.2 to 2.08 mg/g in fresh samples and 2.24 to 59.2 mg/g in dried samples [11,12].

To enhance Fx production, several abiotic factors have been investigated. Among these, nitrogen availability and light are known to play a crucial role, redirecting metabolic flux toward secondary metabolites and regulating photosynthetic pigment composition, with blue and white light reported to promote fucoxanthin accumulation in several microalgal species [9,13,14,15,16,17,18,19]. In addition, multiple studies have demonstrated that different nitrogen sources significantly affect Fx accumulation, revealing a strong correlation across various microalgal species [15,16,20,21,22]. Furthermore, it is well established that light intensity regulates pigment levels, as under low light algae enhance fucoxanthin production to optimize light harvesting, whereas sustained high light typically reduces fucoxanthin levels as cells prioritize photoprotection [14,17,23,24,25,26,27].

However, in microalgae, many steps in the carotenoid biosynthetic pathway remain uncharacterized. Considering that several genes encoding enzymes involved in this pathway have been identified through genome sequencing and alignments, many still lack functional annotation. Diatom species are known to utilize the xanthophyll cycle for photoprotection, particularly the violaxanthin (Vaz) and diadinoxanthin (Ddx) cycles. These cycles help prevent photoinhibition and oxidative damage caused by high light intensity by converting epoxidized carotenoids into de-epoxidized forms, and their regulation is light-dependent [1]. Otherwise, the last steps of Fx biosynthesis remain uncertain. Recent studies proposed that different enzyme isoforms are related to the downstream steps: Violaxanthin de-epoxidase like 2 (VDL2), which transforms diadinoxanthin into alloxanthin, and Zeaxanthin epoxidase 1 (ZEP1), which converts haptaxanthin into phaneroxanthin [28,29]. Understanding the complete xanthophyll biosynthetic pathway in diatoms is crucial for advancing research and developing transgenic organisms with elevated xanthophyll levels, which could have broad applications in biotechnology [29,30,31,32].

Considering the relevance of finding new sources of Fx, our research investigated the centric diatom *Thalassiosira rotula* as a promising candidate for fucoxanthin production. In addition, the rapid growth of *T. rotula*, together with its fully annotated genome and the richness in bioactive compounds—such as polyunsaturated fatty acids, phytosterols, and antioxidant molecules—makes this diatom as a promising platform for biotechnological exploitation within an integrated biorefinery approach [33,34,35,36,37,38,39].

This study aimed to investigate how nitrate availability and light conditions influence Fx metabolism in *T. rotula*. Specifically, we evaluated Fx content under different culture conditions (control, nitrate enrichment, low light, and combination of the two treatments). A comparison between spectrophotometric and HPLC methods was carried out with the aim of proposing, for *T. rotula*, a rapid and practical approach for fucoxanthin quantification. Additionally, the expression of key biosynthetic genes (*PSY*, *PDS*, *ZDS*, *CRTISO*) and xanthophyll cycle (*ZEP*, *VDL*, *DDE*) genes was analyzed to link pigment accumulation with transcriptional regulation, offering insights for future metabolic engineering strategies.

## 2. Results

### 2.1. Growth Dynamic and Morphophysiological Analyses

**Figure 1:**
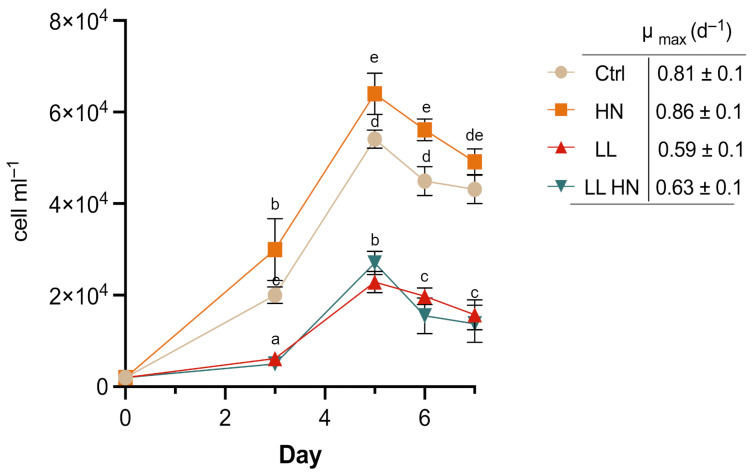
Growth curves of *Thalassiosira rotula Na90A1* under control conditions (Ctrl), nitrate enrichment (HN), low light (LL), and the combined treatment (LL HN). The maximum growth rate (µ_max_) is also shown. Different letters denote statistically significant differences (ANOVA followed by a Tukey’s test, *p* < 0.05). Values represent mean ± SD (n = 3).

The effect of high nitrate supplementation in the medium (HN), low light conditions (LL) and their combination (LL HN) on the growth dynamic of *T. rotula Na90A1* were evaluated under control conditions (Ctrl) and high nitrate supplementation (HN), *T. rotula* exhibited rapid exponential growth from days 3 to 5, reaching a peak on day 5 (late exponential phase). In contrast, cultures under low light (LL) and combined treatment (LL HN) displayed an initial early phase during days 0–3, followed by an exponential growth phase from days 3 to 5, also peaking at day 5. All of the cultures subsequently entered a declining phase from days 5 to 7 (Figure 1).

The HN supplementation significantly enhanced the cell density compared to the control and other treatments, and yielded the highest µ_max_ (Figure 1).

**Figure 2:**
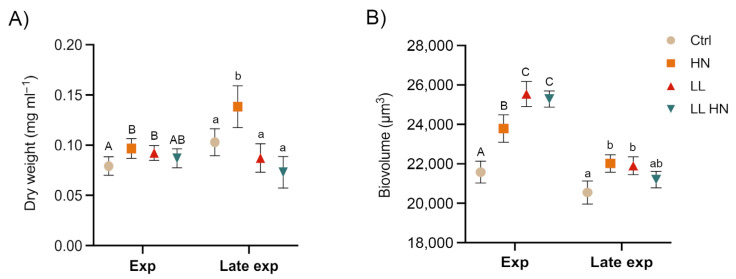
Dry weight (DW mg mL^−1^) (**A**) and biovolume (**B**) (µm^3^) measured at Exp and Late exp of *Thalassiosira. rotula Na90A1* in control (Ctrl) condition, high nitrate (HN) medium, low light condition (LL), and in a combination of both conditions (HN LL). Uppercase letters denote significant differences in the Exp phase; lowercase letters denote significant differences in the Late exp phase (Tukey’s test, *p* < 0.05). Data represent mean ± SD (n = 3).

Biomass and biovolume were measured at the exponential (Exp, day3) and at the late exponential (Late exp, day 5) phase. At the exponential phase, biomass concentration, expressed as dry weight (mg mL^−1^), increased in HN and LL treatments, despite the relatively low cell densities observed under LL (Figure 1 and Figure 2A). The highest biomass concentration was obtained in HN at the late exponential (Late exp) phase (Figure 2A). As cell biovolume, all treatments promoted an increase at the Exp phase, while at the Late exp phase, HN enhanced biovolume compared with the control, reaching values comparable to those under LL and LL HN (Figure 2B).

### 2.2. Effects of the Treatments on the Fucoxanthin Content

**Figure 3:**
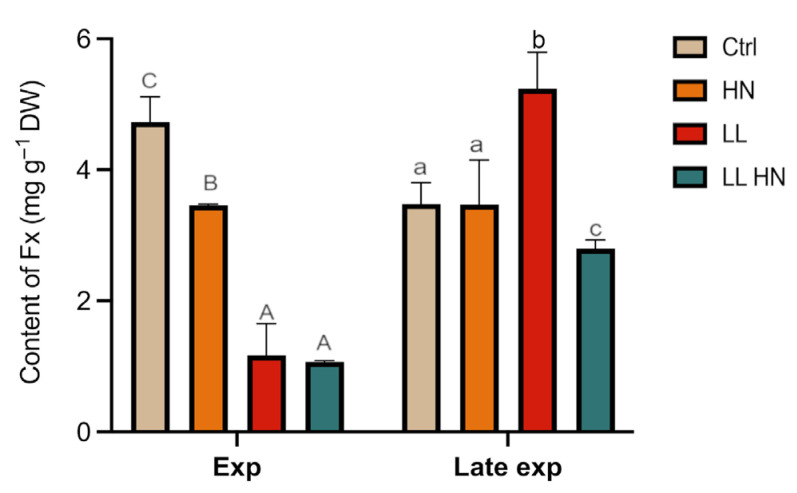
Fucoxanthin content (mg g^−1^ DW) in *Thalassiosira rotula Na90A1*, measured by HPLC in Exp and Late exp phases, under control (Ctrl), high nitrate medium (HN), low light (LL), and the combined (LL HN) treatment. Uppercase letters indicate significant differences in the Exp phase; lowercase letters indicate significant differences in the Late exp phase (Tukey’s test, *p* < 0.05). Data are the mean ± SD (n = 3).

HPLC-based quantification of the major photosynthetic pigments in *T. rotula* Na90A1 at exponential (day 3) and late exponential phases (day 5) is shown in Figure S1. Focusing on fucoxanthin (Fx), the content in the control culture during the exponential phase showed a value of 4.7 mg g^−1^ DW (Figure 3). Overall, the treatments did not induce an increase in Fx level, except under LL at Late exp phase where Fx reached 5.2 ± 0.8 µg mg^−1^ DW. In contrast, Fx levels in HN-treated cultures remained relatively constant across both phases (3.4 ± 0.3 µg mg^−1^ DW) (Figure 3).

### 2.3. Rapid Spectrophotometric Assay to Determine Fx Content in Thalassiosira rotula

We evaluated, by a rapid spectrophotometric approach, the content of Fx in the *T. rotula Na90A1* strain. We focused on the late exponential phase (day 5) for our experiments, since cultures at this stage are characterized by peak density and biomass, providing optimal conditions for subsequent analyses.

Wang et al. [40] applied Equation (1) to calculate fucoxanthin concentration [Fx] mg L^−1^. The parameters *n*_1_ and *n*_2_ account for interference from other pigments and/or cell debris. These correction factors were determined through a regression line, obtained by plotting the absorbance value of the algal residue pigment mixture after ethanol extraction (Algal Suspension Ethanol-ASE) at 445 nm and 663 nm on the x-axis, against the absorbance data of cell debris (Algal Suspension Culture, ASC) at 750 nm on the y-axis.

[Fx] (mg/L) = 6.39 × (A445 − *n*_1_) − 5.18 × (A663 − *n*_2_) (1)

**Figure 4:**
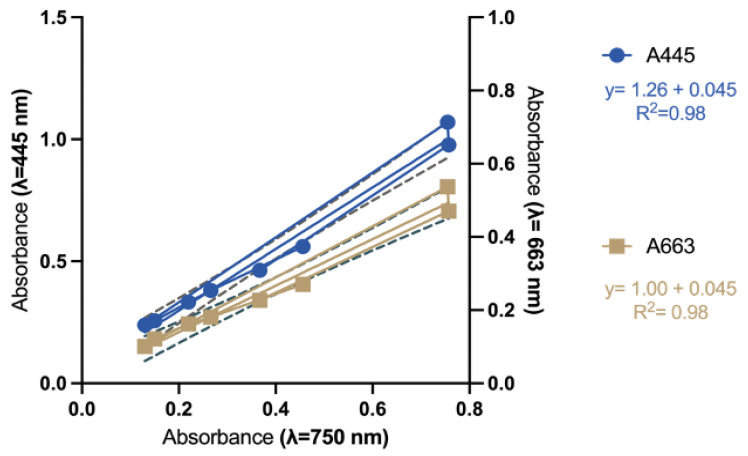
Absorbance of the residue pigment mixture and cell debris at 445 nm and 663 nm. The x-axis shows the absorbance of cell culture at 750 nm. The blue circle indicates A445, and the brown square indicates A663. Fitted curves from three replicates per wavelength are shown as blue and brown lines, respectively. Dashed lines represent 95% of confident interval (CI).

Based on the “background noise” as a function of the number and the type of cells used to make the measurements, we considered it appropriate to recalculate *n*_1_ and *n*_2_ in Equation (1) for *T. rotula*. So, we measured before the absorbance of algal culture (ASC) at 750 nm and after at 445 nm and 663 nm, following pigments extraction and resuspending the saved cell debris in ethanol (ASE). By plotting the absorption values of ASE at 445 nm and 663 nm as the x-axis, and ASC measured at 750 nm represented on the y-axis, we obtained the regression lines (Figure 4). As reported in [40], *n*_1_ and *n*_2_ correspond to the equation of the regression line (Figure 4).

So, for *T. rotula*, *n*_1_ and *n*_2_ were as follows:

*n*_1_ (A445′) = 1.26 × A750 + 0.045

*n*_2_ (A663′) = 1.00 × A750 + 0.01

Subsequently, the coefficients *n*_1_ and *n*_2_ were replaced into Equation (1), resulting in the derivation of Equation (2), further simplified into Equation (3), which provides a corrected formulation for the estimation of Fx concentration in *T. rotula*:

[Fx]’ (mg/L) = 6.39 × (A445 − 1.26 × A750 + 0.045) − 5.18 × (A663 − 1.00 × A750 + 0.01)(2)

[Fx]’ (mg/L) = 6.39 × A445 − 5.18 × A663 − 2.87 × A750 − 0.24(3)

**Figure 5:**
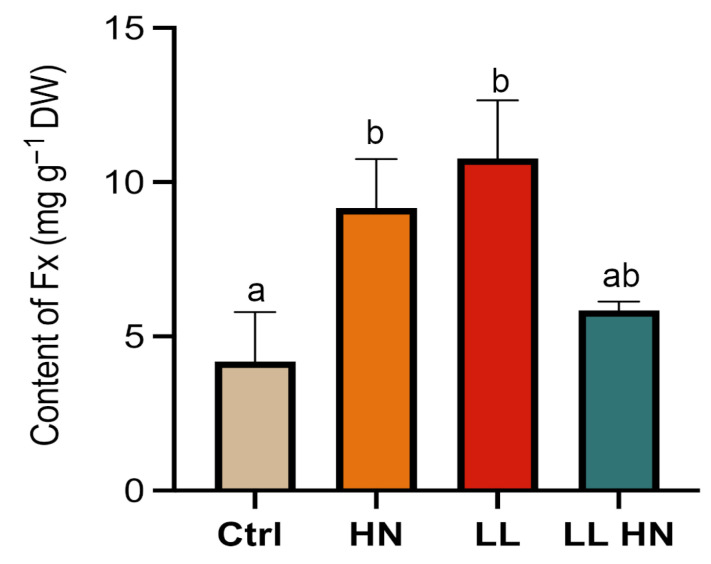
Fucoxanthin content (mg g^−1^ DW) in *Thalassiosira rotula Na90A1*, at the Late exp phase measured by spectrophotometric method under control (Ctrl), high nitrate medium (HN), low light (LL), and the combined (LL HN) treatments. Different letters indicate significant differences among the treatments (ANOVA, Tukey’s test, *p* < 0.05). Values represent mean ± SD (n = 3).

By applying Equation (3), the Fx content measured during the Late exp phase using the spectrophotometric method reached its maximum value of 10.8 ± 0.9 mg g^−1^ DW under the LL treatment, approximately twice the value observed in the control (Figure 5).

### 2.4. Fucoxanthin Quantification: HPLC Versus Spectrophotometric Analysis

When comparing spectrophotometric values with those obtained with the HPLC (Table 1), an overestimation of Fx content was founded in all the treatments. In fact, the agreement between the two methods (% error) ranged from 14% in the control to 164% in the HN treatment. Despite being optimized for *T. rotula*, the new formula yielded results comparable with well-established HPLC method only under control conditions.

### 2.5. Expression Patterns of Key Genes Involved in the Fucoxanthin Biosynthetic Pathway

**Figure 6:**
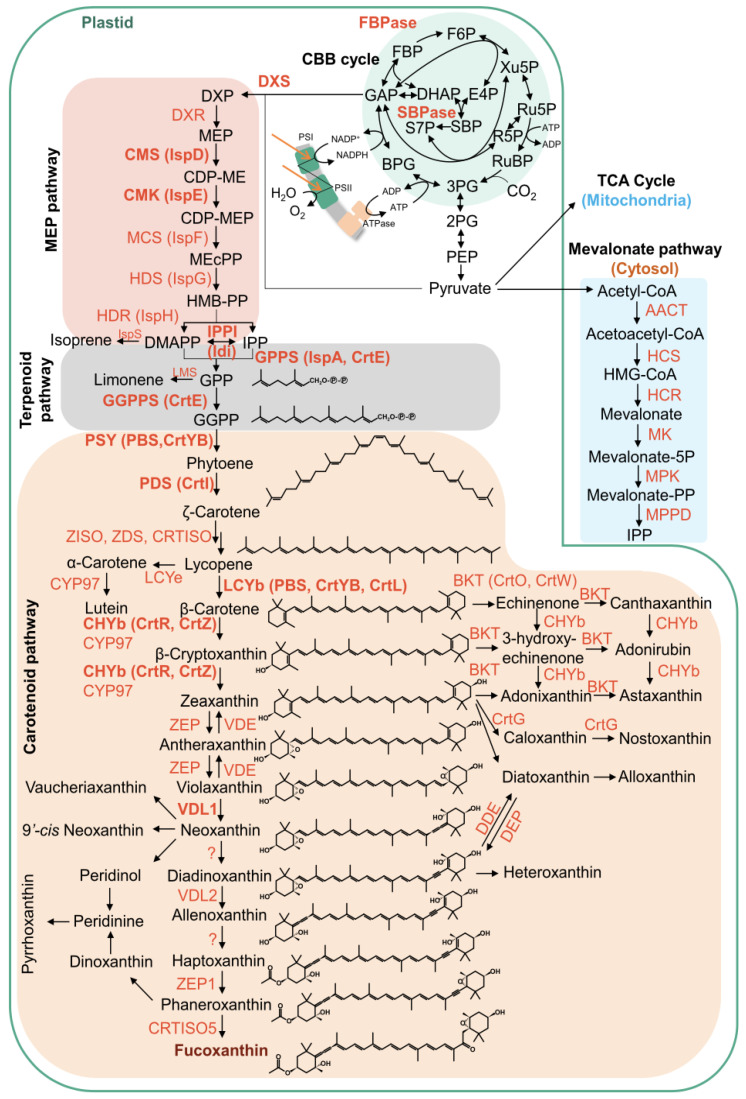
Schematic representation of fucoxanthin biosynthetic pathways and precursors. Image adapted from Tanaka et al. [29]. Abbreviations: AACT, acetoacetyl-CoA thiolase; BKT, beta-carotenoid ketolase; BPG, 1,3-bisphosphoglycerate; CBB, Calvin–Benson–Bassham; CDP-ME, 4-diphosphocytidyl-2-C-methylerythritol; CDP-MEP, 4-diphosphocytidyl-2-C-methyl-d-erythritol 2-phosphate; CHYb, beta-carotenoid hydroxylase; CMK, 4-diphosphocytidyl-2-C-methyl-d-erythritol kinase; CMS, 2-C-methyl-d-erythritol 4-phosphate cytidylyltransferase; CRTISO, carotenoid isomerase; CYP97, cytochrome P450 hydroxylase; DDE, diadinoxanthin de-epoxidase; DHAP, dihydroxyacetone phosphate; DEP, diatoxanthin epoxidase; DMAPP, dimethylallyl pyrophosphate; DXR, 1-deoxy-d-xylulose 5-phosphate reductoisomerase; DXP, 1-deoxy-d-xylulose 5-phosphate; DXS, 1-deoxy-d-xylulose 5-phosphate synthase; E4P, erythrose 4-phosphate; FBP, fructose 1,6-bisphosphate; F6P, fructose 6-phosphate; GAP, glyceraldehyde 3-phosphate; GGPP, geranylgeranyl diphosphate; GGPPS, geranylgeranyl diphosphate synthase; GPP, geranyl diphosphate; GPPS, geranyl diphosphate synthase; HCR, HMG-CoA reductase; HCS, hydroxymethylglutaryl-CoA synthase; HDR, 4-hydroxy-3-methylbut-2-en-1-yl diphosphate reductase; HDS, 4-hydroxy-3-methylbut-2-en-1-yl diphosphate synthase; HGM-CoA, 3-hydroxy-3-methylglutaryl-CoA; HMB-PP, (E)-4-hydroxy-3-methylbut-2-enyl pyrophosphate; IPP, isopente- nyl pyrophosphate; IPPI, isopentenyl-diphosphate isomerase; IspS, isoprene synthase; LCYb, lycopene beta cyclase; LCYe, lycopene epsilon cyclase; MCS, 2-C-methyl-d-erythritol 2,4-cyclodiphosphate synthase; LMS, limonene synthase; MEcPP, 2-C-methyl-d-erythritol 2,4-cyclodiphosphate; MEP, 2-C-methylerythritol 4-phosphate; MK, mevalonate-5-kinase; MPK, phosphomevalonate kinase; MPPD, mevalonate-5-pyrophosphate decarboxylase; NXS, neoxanthin synthase; PDS, phytoene desaturase; PEP, phosphoenolpyruvate; PSI, photosystem I; PSII, photosystem II; PSY, phytoene synthase; 2PG, 2-phosphoglycerate; 3PG, 3-phosphoglycerate; RuBP, ribulose 1,5-bisphosphate; R5P, ribose 5-phosphate; Ru5P, ribulose 5-phosphate; SBP, sedoheptulose 1,7-bisphosphate; S7P, sedoheptulose 7-phosphate; TCA, tricarboxylic acid; VDE, violaxanthin de-epoxidase; VDL, violaxanthin de-epoxidase-like; Xu5P, xylulose 5-phosphate; ZDS, zeta-carotene desaturase; ZEP, zeaxanthin epoxidase; ZISO, zeta-carotene isomerase.

To better understand how the experimental conditions influenced the biosynthesis pattern of Fx, we analyzed the expression levels of selected genes involved in the Fx biosynthetic pathway (Figure 6).

Genes expression was assessed in both the exponential (Exp) and late exponential (Late exp) phases.

**Figure 7:**
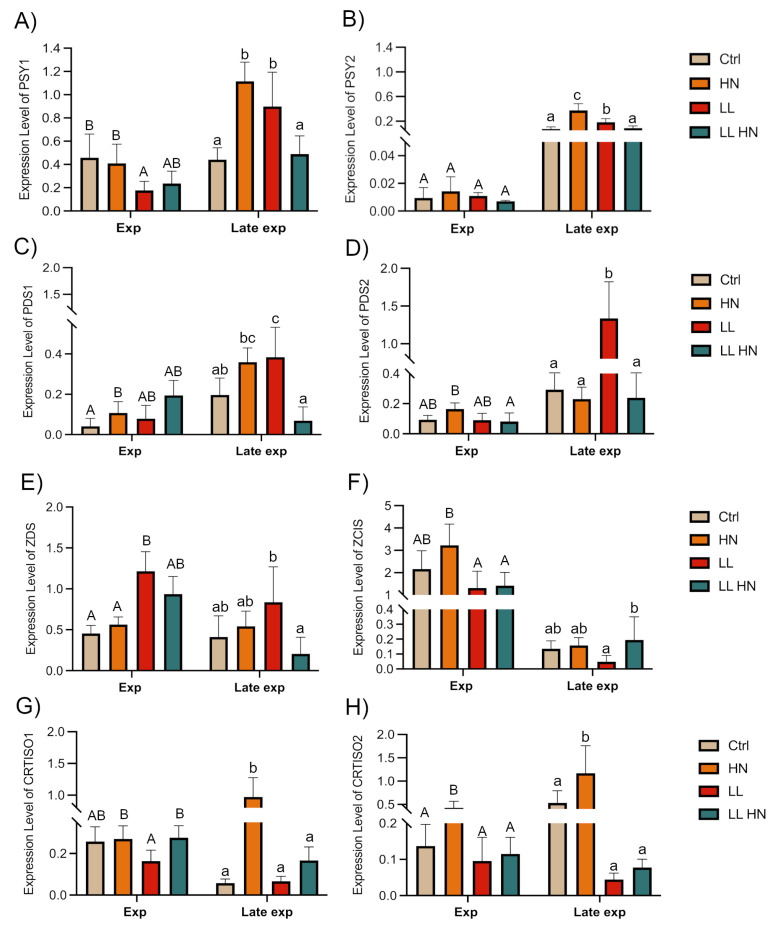
Expression level of key genes involved in the carotenoid biosynthesis pathway in *Thalassiosira rotula Na90A1* in Exp and Late exp phases. Cultures were grown under standard medium (Ctrl), high nitrate medium (HN), low light condition (LL), and in a combination of both conditions (LL HN). Abbreviations: *PSY*, *phytoene synthase* (**A**,**B**); *PDS*, *phytoene desaturase* (**C**,**D**); *ZCIS*, *15-cis-ζ-carotene isomerase* (**E**); *ZDS*, *ζ-carotene desaturases* (**F**); *CRTISO*, *carotenoid isomerase* (**G**,**H**). Uppercase letters indicate significant differences in the Exp phase; lowercase letters indicate significant differences in the Late exp phase (Tukey’s test, *p* < 0.05). Data were analyzed using the 2^−ΔCt^ method and represent the mean ± SD; (n = 3).

**Figure 8:**
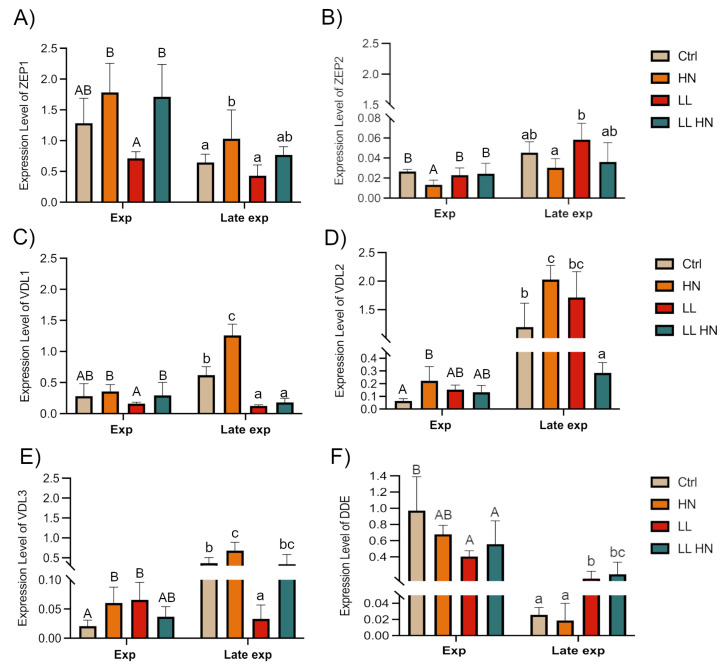
Expression level of key genes involved in the xanthophyll cycle in *Thalassiosira rotula Na90A1* in Exp and Late exp phases. Cultures were grown under standard medium (Ctrl), high nitrate medium (HN), low light condition (LL), and in a combination of both conditions (LL HN). Abbreviations: *ZEP*, *zeaxanthin epoxidase* (**A**,**B**); *VDL*, *violaxanthin de-epoxidase like* (**C**–**E**); *DDE*, *diadinoxanthin de-epoxidase* (**F**). Uppercase letters indicate significant differences in the Exp phase; lowercase letters indicate significant differences in the Late exp phase (Tukey’s test, *p* < 0.05). Data were analyzed using the 2^−ΔCt^ method and represent the mean ± SD; (n = 3).

Globally, our results showed that the analyzed key genes exhibited markedly different expression patterns (Figure 7 and Figure 8). The two *PSY* isoforms (*PSY1* and *PYS2*), responsible for phytoene synthesis in the first step of the pathway, displayed a significant upregulation under HN and LL conditions in the Late exp phase (Figure 7A,B). On the other hand, *PDS* isoforms (encoding for a phytoene desaturase) reached their highest expression under LL condition in the Late exp phase (Figure 7C,D).

In the subsequent step of the biosynthetic pathway, *ZDS* (ζ-carotene desaturases) overexpression was mostly observed under LL condition in both phases (Figure 7E). In contrast, *ZCIS* (coding for a 15-cis-ζ-carotene isomerase) showed higher expression in the Exp phase under HN condition, but decreased in the late exponential phase, except under LL HN treatment (Figure 7F). Regarding the two *CRTISO* isoforms (coding for the carotenoid isomerase), *CRTISO1* showed a higher expression in the Exp phase in all treatments except for the LL, while in Late exp, the HN treatment induced a significant rise (Figure 7G); conversely, *CRTISO2* showed a peak of expression in HN condition in both growth phases (Figure 7H).

Furthermore, we focused on the genes of the xanthophyll cycle, *ZEP* (*zeaxanthin epoxidase*) and *VDL* (*violaxanthin de-epoxidase-like*). *ZEP1* exhibited higher expression under HN and LL HN conditions in both growth phases (Figure 8A); *ZEP2* was expressed at similar levels at the Exp phase in all treatments, except HN and with a similar trend in Late exp phase (Figure 8B). Regarding the *violaxanthin de-epoxidase like* (*VDL*) genes, phylogenetic analysis of putative sequences of *T. rotula*, aligned with the recent annotations [32], identified three possible isoforms. Specifically, *VDL1* isoform of *T. rotula* clustered closely to *VDL1* of *Thalassiosira pseudonana* and *Phaeodactylum tricornutum*, whereas *VDL2* and *VDL3* were both closely related to *VDL2* of *T. pseudonana* and *P. tricornutum* (Supplementary Figure S2).

Relative expression analysis showed that *VDL1* exhibited the maximum levels in the Late exp phase under HN conditions, a trend also observed for *VDL3* (Figure 8C–E). At the same time, *VDL2* exhibited a significant increase in the Late exp phase under both HN and LL conditions (Figure 8D).

Finally, the single isoform of *DDE* (*diadinoxanthin de-epoxidase*), a gene involved in the Diadinoxanthin (Ddx) cycle, was investigated, showing the highest expression in Ctrl condition at the Exp phase, whereas in the Late exp, its expression increased under LL and LL HN treatments, compared to the control (Figure 8F).

## 3. Discussion

This study examined how nitrate availability and light conditions influence fucoxanthin content and the expression of key biosynthetic genes in the marine diatom *Thalassiosira rotula*. Considering the central role of fucoxanthin not only as a key player in the photosynthetic process, but also from a biotechnological perspective, our results demonstrate that in *T. rotula* nitrogen and light regimes affect Fx biosynthesis and its accumulation. Our results showed that the high nitrogen availability (HN) significantly enhanced the growth, biomass and biovolume across growth phases, as also reported for *P. tricornutum* [41]. These results indicate that high nitrate supplementation induced positive effects on *T. rotula* growth. Compared with the control and with the LL and LL HN treatments, HN led to a significant increase in cell density, suggesting that nutrient availability was the dominant factor driving biomass accumulation under the tested conditions. Furthermore, the maximum growth rate (µ_max_) was highest under HN, supporting the view that nitrate enrichment enhances not only cell yield but also the overall growth dynamics. This trend is consistent with observations in *Thalassiosira weissflogii*, where nitrate enrichment (8.82 mM NaNO_3_) resulted in an ~18% increase in mean cell volume compared to nitrate-limited cultures [42]. Similarly, in *Skeletonema costatum*, elevated nitrate (10 mM) enhanced cell biovolume by ~20% relative to standard f/2 medium, while simultaneously increasing cellular nitrogen reserve [43], while LL lowers cell density but increases cell size [19,26,44,45,46].

Previous observations in diatoms reported that nutrient and light regimes strongly influence the balance between photoprotection and light-harvesting pigments [33,47]. Our HPLC analysis confirmed that these treatments significantly influenced pigment concentrations. Notably, the Fx concentration, reported yet in the Ctrl in the Exp phase (4.7 mg g^−1^ DW), was higher than values reported for other microalgae, such as *S. costatum* (0.36 mg g^−1^ DW), *Odontella sinesis* (1.18 mg g^−1^ DW), *Nitzschia laevis* (1.68 mg g^−1^ DW), *Chaetoceros gracilis* and *Chaetoceroscalcitrans* (2.24 and 2.33 mg g^−1^ DW, respectively) [11,44,48,49], highlighting the biotechnological potential of our species [11,44,48,49]. In addition, under low-light conditions, at the late exponential growth phase, Fx content seems to increase (5.2 mg g^−1^ dry weight) and/or maintains its content stable. The evidence that *T. rotula* is able to maintain a substantial fucoxanthin (FX) content even in the late exponential phase may indicate its potential suitability as a source for the extraction of additional bioactive metabolites. Indeed, as reported in the literature, several secondary metabolites are typically synthesized during the late stages of growth [37,50,51]. This would facilitate the development of integrated microalgal biorefineries approaches that allow the simultaneous extraction of multiple metabolites, thereby improving process efficiency and resource utilization.

Furthermore, the observation that fucoxanthin content in *T. rotula* is slightly increased at the late exponential phase under low light condition, compared to the control, contrasts with findings in other diatom species, where low irradiance generally promotes Fx accumulation. For instance, in *Cyclotella criptica,* light intensities between 10 and 30 μmol m^−2^ s^−1^ promoted the fucoxanthin accumulation of 33.3% higher than control (0.76%) during the exponential phase, and *P. tricornutum* reached a maximum of 16.03 mg g^−1^ DW at 20 μmol m^−2^ s^−1^ [11,14,44]. Similarly, another study on *P. tricornutum* reported Fx levels of 1.7 mg g^−1^ (fresh weight) under low-light conditions, compared with 0.54 mg g^−1^ (fresh weight) under high-light conditions [19]. This supports that diatoms optimize pigment composition under reduced irradiance to maximize photon capture efficiency (e.g., *P. tricornutum* and *Cyclotella meneghiniana*) [11,26]. Nevertheless, such comparisons should be made cautiously, as pigment dynamics are known to be highly species-specific and strongly influenced by growth conditions. In our case, under low-light conditions, Fx content remains relatively stable, whereas other pigments, notably Chl a and carotenoids, increased significantly (Figure S1). This observation suggests that *T. rotula* may adopt distinct photo-acclimation strategies, involving the modulation of accessory pigments rather than major changes in fucoxanthin content. The stability of Fx content under nitrate enrichment, similar to observations made in *P. tricornutum* [21], may also reflect strain-specific regulatory mechanisms rather than a generalizable response.

Interestingly, in *T. rotula*, high nitrate treatment did not yield the highest Fx content but rather maintained stable pigment levels across growth phases. This finding suggests that in *T. rotula*, nitrate availability may primarily support sustained biosynthetic capacity rather than driving pigment accumulation. Similar results have been observed in other diatoms, where nitrogen sufficiency stabilizes chlorophyll and carotenoid pools, while nitrogen limitation often leads to pigment degradation as cells redirect resources to essential metabolism [22].

In addition, in this work we also tested the possibility of using a spectrophotometric method to quantify the Fx in *T. rotula*. Although we attempted to refine the equation proposed by Wang [40] for Fx spectrophotometric determination, the outcomes between spectrophotometric and HPLC quantification are not directly comparable in absolute values; rather, they show consistency at the level of overall trends only under control conditions (14.0% error compared to HPLC value), whereas stressed cultures showed increasing discrepancies. This pattern is consistent with observations in *P. tricornutum*, where spectrophotometric estimates deviated less than 5%; the same equation applied to other diatoms (*Chaetoceros muelleri* and *Thalassiosira pseudonana*) showed standard error ranging from 3% to approximately 14.6% compared to HPLC values [40]. Differences in pigment composition, cell size, other cellular components, and growth stage may affect the accuracy of the equation [40]. We hypothesize that such differences are mainly attributed to spectral overlap with other carotenoids and/or matrix effects, which can lead to an overestimation of fucoxanthin under non-standard physiological states. Overall, although the proposed spectrophotometric correction for *T. rotula* improves accuracy under control conditions and might be used as a preliminary screening tool to estimate Fx content under standard condition, its limited consistency under stress treatments, suggests that this approach is not suitable for large-scale industrial application. Despite its potential to reduce processing time HPLC remains the most reliable and accurate method for fucoxanthin quantification in *T. rotula*. Further methodological refinements and validation across different culture conditions would be required before the spectrophotometry approach can be considered a robust alternative.

In light of the fact that Fx production is a complex and fine-tuned process, influenced by various factors [1,19,28], the expression levels of the key genes involved in the carotenoid pathways at the Exp and Late exp phases in all culture conditions were investigated.

Our finding as upregulation of most biosynthetic genes examined under low light, is consistent with the need to expand the antenna complex, whereas modulation of xanthophyll cycle genes reflects adjustments in photoprotective capacity. Firstly, the high expression of *PSY* and *PDS* genes, regarded as a rate-limiting step in carotenoid biosynthesis, align with the other studies conducted on *P. tricornutum* [1,14,19,26]. Moreover, a key role is mediated by the violaxanthin cycle, starting with the formation of zeaxanthin from ß-car (Figure 8). The expression levels of different isoforms have shown that *ZEP1* was upregulated by HN and LL HN in both phases, confirming its relevant role; on the other hand, *ZEP2* was highly expressed under LL condition, although its expression was much lower than *ZEP1*. These results highlight distinct functional roles for the isoforms. According to recent studies conducted in *P. tricornutum*, *ZEP1* encodes an enzyme to convert the haptoxanthin in phaneroxanthin, the last precursor of Fx. The roles of *ZEP2* and *ZEP3* remain uncertain but likely involve the conversion of zeaxanthin to violaxanthin and are strongly regulated by light intensity (Figure 6) [26,52,53]. Other studies validated the role of *VDL* isoforms in diatoms [28,29,32]. In *T. rotula*, the *VDL* isoforms exhibited different expression levels related to growth conditions: in the Exp phase, a rise of the expression level in response to the different treatments vs. Ctrl was seen; nonetheless, in the Late exp the highest expression levels were observed only in HN, except for *VDL2* with the highest level in LL condition. This differential expression likely reflects distinct isoform functions; as reported by [28], VDL1 catalyzes the conversion of violaxanthin to neoxanthin, the early product of Fx biosynthesis, while VDL2 converts diadinoxanthin to allenoxanthin (Figure 6).

Overall, these results highlight the synergistic role of light and nutrients in optimizing fucoxanthin production and regulating carotenoid metabolism in *T. rotula*. Our findings suggest that manipulating environmental conditions, such as light and nutrient availability, could be a viable strategy to enhance fucoxanthin yields in *T. rotula* and proposing it as new candidate for fucoxanthin production. Such insights provide a useful basis for optimizing large-scale cultivation protocols, although further validation across different strains and growth systems will be required before industrial application. Finally, gene expression patterns reveal condition-dependent regulation of Fx biosynthesis and xanthophyll cycle genes, underscoring the complexity of carotenoid regulation in diatoms and also providing a set of potential molecular targets for metabolic engineering aimed at further enhancing Fx biosynthesis.

## 4. Materials and Methods

### 4.1. Microalgal Strain and Growth Conditions

Marine diatom *Thalassiosira rotula* Na90A1 was kindly provided by Culture Collection of the Zoological Station “Anton Dorn” (Naples, Italy). A preculture volume (250 mL) at the initial cell density of 10^3^ cell mL^−1^ was grown in f/2 medium [54] at a constant temperature of 19 °C under fluorescent lamp illumination (80 μmol m^−2^ s^−1^) with a 12:12 h light/dark cycle. At the exponential phase (5 days), the preculture was transferred into fresh medium in a 2 L Erlenmeyer flask (working volume 1.5 L) with mixing and aeration by bubbling air. Cultures were grown for 8 days under three distinct conditions: (i) high nitrate supplementation (HN, 8.82 mM NaNO_3_), (ii) low light intensity (LL, 30 μmol m^−2^ s^−1^), and (iii) a combination of both treatments (LL HN, 30 μmol m^−2^ s^−1^ and 8.82 mM NaNO_3_). The control cultures (Ctrl) were grown under standard conditions in f/2 medium containing 882 μM NaNO_3_ at 80 μmol m^−2^ s^−1^. All experiments were performed in triplicate.

### 4.2. Growth Dynamic and Morphophysiological Analyses

All of the cultures subsamples at days 3, 5, 6, 7 were fixed in Lugol’s iodine solution (Sigma, Darmstadt, Germany) and cell density was determined by counting samples in a Sedgewick Rafter chamber under a Leica DMI microscope (Leica, Milan, Italy) at 100× magnification. The maximum growth rate (µ_max_) was calculated as μ = (ln N_1_) − ln (N_0_)/(t_1_ − t_0_), where N_0_ and N_1_ represent the cell densities (cells/mL) at t_0_ (beginning of the exponential phase) and t_1_ (end of the exponential phase when ln(N) versus time was linear), respectively.

For all the cultures, biomass determination was performed at the exponential growth phase (Exp, days 3) and at the late exponential growth phase (Late exp, day5), corresponding to the pick of the exponential growth. (Briefly, 100 mL of cultures were filtered using the Whatman GF/C filter, dried at 80 °C for 24 h and the quantified as dry weight per milliliter (DW mg mL^−1^).

For morphometric analysis, to calculate biovolume, images of cells at Exp and Late exp phases were taken under a Leica DMRB microscope equipped with a digital camera Leica DFC320 (Leica, Milan, Italy) at 400× magnification. The perivalvar and apical size were measured using Fiji software 2.16 (https://imagej.net/software/fiji/ (accessed on 15 October 2024)), and biovolume was calculated according to Sun and Liu [55].

### 4.3. Fucoxanthin Quantification by HPLC Analysis

For pigment content using HPLC, *T. rotula* cultures at Exp (day 3) and Late exp (day 5) phases were filtered on Glass Fiber Filter (G/FF) and frozen in liquid nitrogen. For pigment extraction, 3 mL methanol was added to freeze-dried filters. The pigment solution was filtered through a 0.22 μm nylon syringe filter (VWR, Radnor, PA, USA). Then, 250 μL of an Ion Pairing Agent (ammonium acetate 1 mol L^−1^, final concentration 0.33 mol L^−1^) was added to 500 μL of the pigment extract and incubated for 5 min in darkness at 4° C. The ion pairing agent was used to increase pigment hydrophobicity in order to obtain a better retainment on the column improving the peaks quality. The collected pigment solutions were analyzed at 440 nm using a Hewlett Packard photodiode array detector model DAD series 1100 (HPLC-DAD), which gives the 400–700 nm spectrum for each detected pigment. A fluorometer (Hewlett Packard standard FLD cell series 1100, Wilmington, NC, USA) with excitation at 407 nm and emission at 665 nm allowed the detection of fluorescent molecules (chlorophylls and their degraded products). The reversed-phase column corresponded to an apolar stationary phase composed of silica beads possessing aliphatic chains of 8 carbon atoms (2.6 mm diameter C8 Kinetex column; 50 mm × 4.6 mm; Phenomenex^®^, Torrance, CA, USA). The mobile phase was composed of two solvent mixtures: A, methanol: aqueous ammonium acetate (1 M) (70:30, *v*/*v*) and B, absolute methanol. During the 12 min elution, the gradient between the solvents was programmed: 75% A (0 min), 50% A (1 min), 0% A (8 min), 0% A (11 min), and 75% A (12 min).

The samples were analyzed to identify pigments based on retention time, absorption spectra, and co-chromatography through the pigment standards purchased from DHI (Hørsholm, Denmark).

The content of pigments in all samples was calculated using the following formula:

µg/µL = (Area × Coeff. × V. Extr.)/(V. Iniet. × V. Filtr. × 0.5/0.75) × 1000

in which:

Area = area of picks (DAD);
Coeff. = coefficient of pigment;
V. Extr., Iniet., Filtr. = volume of samples extracted, injected and filtered, respectively.

### 4.4. Extraction and Spectrophotometric Determination of Fucoxanthin

For a rapid spectrophotometry fucoxanthin assay the protocol of Wang et al. [40] with some modifications was applied.

Fucoxanthin concentration in *T. rotula* was determined spectrophotometrically (Cary 60UV-VIS Spectrophotometer, Agilent, Santa Clara, CA, USA) following the protocol of Wang et al. [40] with modifications. Briefly, 50 mL of exponential-phase cultures were centrifuged (4700× *g*, 15 min). The pellet was resuspended in f/2 medium (Algal Suspension Culture, ASC) (A750 ranges from 0.1 to 0.8). In parallel, 50 mL of cultures were centrifuged (4700× *g*, 15 min), and pellets resuspended in an equal volume of ethanol (Algal Suspension Ethanol, ASE) (A445 and A663 range from 0.2 to 1). Three independent biological samples and three technical replicates per sample were performed, all within 5 min of sample preparation.

Fucoxanthin concentration (Fx, mg/L) was calculated using Equation (1) [40], with modifications. Specifically, the correction factors *n*_1_ and *n*_2_, accounting for interference from residual pigments in the ethanol suspension (ASE) and from cell debris in the culture suspension (ASC), were derived from absorbance at 445 nm and 663 nm relative to A_750_. medium. These correction factors were determined for *T. rotula* and applied in Equation (1), resulting in the Equation (2), further semplified in Equation (3).

### 4.5. RNA Extraction and Quantitative Real-Time PCR (RT-qPCR)

To evaluate the expression levels of key genes in Fx biosynthesis in different culture conditions, culture pellets at Exp and Late exp phases were collected using a centrifuge 14,000 rpm × 30 min. Total RNA was carried out using the RNA Isolation Mini Kit (Agilent Technologies, Santa Clara, CA, USA) protocol. Additional steps, after the addition of extraction buffer and vortexing, were introduced to remove proteins: supernatant obtained after a centrifugation at full speed (13,000 rpm × 5 min), were transferred to a clean Eppendorf and an equal volume of chloroform: isoamyl alcohol (24:1) was added, vortexing vigorously again, and then finally centrifuged at full speed (13,000 rpm × 15 min). Each RNA sample was treated with RNAse-free DNAse (Qiagen, Hilden, Germany), to further purify the solution.

The RNA extracted was assessed for purity and concentration using a NanoDrop (ND-1000 UV-VIS spectrophotometer; NanoDrop Technologies, Wilmington, DE, USA). The integrity of total RNA was checked by agarose gel electrophoresis.

RNA samples were converted into cDNA through reverse transcription using the SuperScript III First-Strand Synthesis System for RT-PCR (Invitrogen, Carlsbad, CA, USA), following the manufacturer’s guidelines. The gene expression analyses were performed using specific primer pairs designed using Primer3 [56,57,58] (Supplementary Table S1), and the efficiency of the primers was determined from five serial dilutions of mixed cDNAs using the formula: E = 10^(−1/slope) [34]. The Reverse transcription-quantitative PCR (RT-qPCR) experiments were conducted using a MicroAmp Optical 384-Well Reaction Plate (Applied Biosystems, Foster City, CA, USA) on a ViiA™ 7 Real-Time PCR System (Applied Biosystems, Foster City, CA, USA). Each PCR reaction had a total volume of 10 μL, consisting of 5 μL of Power SYBR Green PCR Master Mix 2X (Applied Biosystem, Monza, Italy), 1 μL of cDNA template, and 4 μL of 0.7 μM oligo mix (forward and reverse).

The normalized expression levels of each gene of interest relative to the most stable reference genes, RPS, TUBa and GAPDH, were calculated [34]. The obtained results were analyzed according to the 2^−ΔCt^ method [34].

### 4.6. Statistical Analysis

For each condition, three Erlenmeyer flasks were employed as biological replicates. For morphometric analysis, a minimum of 70 cells were measured. All data are expressed as mean ± standard deviation of biological replicates. Statistical analyses, including one-way and two-way analysis of variance (ANOVA), followed by a Tukey’s test, were performed using the statistical software GraphPad PRISM 9 (GraphPad Software Inc., San Diego, CA, USA). Statistically significant differences were considered at *p* < 0.05.

## 5. Conclusions

In conclusion, our study highlights how nitrate availability and light conditions interact to regulate fucoxanthin production in *Thalassiosira rotula*. High nitrate supply clearly stimulated growth, biomass, and biovolume, confirming the central role of nutrient availability in sustaining diatom proliferation. By contrast, low-light conditions favored a slight increase in fucoxanthin compared with the control at the late exponential phase, reflecting the need to optimize pigment composition for efficient light harvesting under reduced irradiance. Interestingly, nitrate enrichment alone stabilized fucoxanthin levels across growth phases, suggesting that its main role is to maintain pigment biosynthesis rather than to actively promote accumulation. At the transcriptional level, the modulation of key biosynthetic genes (*PSY*, *PDS*, *ZDS*, *CRTISO*) together with xanthophyll cycle genes (*ZEP*, *VDL*, *DDE*) revealed condition-specific regulatory patterns, with clear evidence of isoform-dependent responses. These findings point to a multilayered regulation of fucoxanthin metabolism, where light and nutrients act synergistically to fine-tune pigment synthesis and photoprotection. Altogether, these findings highlight the synergistic role of nutrients and light in shaping fucoxanthin production and proposing *T. rotula* as a new candidate for fucoxanthin production, providing a useful basis for optimizing cultivation strategies and exploring molecular targets for biotechnological applications.

## Figures and Tables

**Table 1:** Comparison of Fucoxanthin content (mg g^−1^ DW) in *Thalassiosira rotula Na90A1* at the Late exp phase, measured by HPLC method and spectrophotometry (Spectro), under control (Ctrl), high nitrate medium (HN), low light (LL), and the combined (LL HN) treatments. Statistical differences were determined using ANOVA followed by a Tukey’s ranked test (* *p* < 0.05). Values are mean ± SD (n = 3).

	HPLC (mg g^−1^ DW)	Spectro (mg g^−1^ DW)	Error (%)
Ctrl	3.63 ± 0.5	4.14 ± 0.7	14 ± 0.3
HN	3.47 ± 0.8 *	9.17 ± 0.9	164 ± 1.5
LL	5.2 ± 0.9 *	10.77 ± 0.8	79 ± 0.5
LL HN	2.79 ± 0.5 *	5.85 ± 0.6	52 ± 0.9

## Data Availability

Data are contained within the article and Supplementary Materials.

## Supplementary Materials

The following supporting information can be downloaded at: https://www.mdpi.com/article/10.3390/plants14213344/s1, Figure S1: Photosynthetic pigments content (mg g−1 DW) of T. rotula Na90A1 strain measured using the HPLC method in Exp and Late exp phases. Abbreviations: Chl a, Chlorophyll a (A); Chl c, Chlorophyll c (B); ß-car, Beta-carotene (C); Vx, Violaxanthin (D); Ddx, Diadinoxanthin (E); Dtx, Diatoxanthin (F). Cultures were grown under standard medium (Ctrl), high nitrate medium (HN), low light condition (LL), and in a combination of both conditions (LL HN). Up-percase letters indicate significant differences in the Exp phase; lowercase letters indicate significant differences in the Late exp phase (Tukey’s test, *p* < 0.05). Data are the mean ± SE; n = 3. Figure S2: Phylogenetic analysis of predicted Violaxanthin de-epoxidase-like (VDL) isoforms. The three predicted VDL isoforms from Thalassiosira rotula were identified using Unipro UGENE 51.0 and annotated via the NCBI BLAST tool (2.17.0). Sequence alignment was performed with the MUSCLE algorithm (integrated in Unipro UGENE 51.0) using the most similar VDLs from various diatom species. A phylogenetic tree was generated with the Maximum Likelihood method and 1000 bootstrap replicates using MEGA11. The VDL isoforms from T. rotula are indicated with red circles. Phylogram was constructed by the neighbor-joining method using the K2P distance protocol in MEGA ver.5.0. Numerical value at the nodes of the branches indicates bootstrap values and asterisks indicate species studied in this study; Table S1: Primers used in the present study.
