# Supplementary material for: Influence of Nitrate and Light on Fucoxanthin Content and Key Gene Expression in the Marine Diatom Thalassiosira rotula
Source: Plants (Basel). 2025 Oct 31;14(21):3344. doi: 10.3390/plants14213344 (PMC12610687; doi:10.3390/plants14213344)

Figure S1: Photosynthetic pigments content (mg g^{-1} DW) of *T. rotula Na90A1* strain measured using the HPLC method in Exp and Late exp phases. Abbreviations: Chl a, Chlorophyll a (A); Chl c, Chlorophyll c (B); ß-car, Beta-carotene (C); Vx, Violaxanthin (D); Ddx, Diadinoxanthin (E); Dtx, Diatoxanthin (F). Cultures were grown under standard medium (Ctrl), High Nitrate medium (HN), low light condition (LL), and in a combination of both conditions (LL HN). Up-percase letters indicate significant differences in the Exp phase; lowercase letters indicate significant differences in the Late Exp phase (Tukey's test, $p < 0.05$). Data are the mean ± SD; n=3.

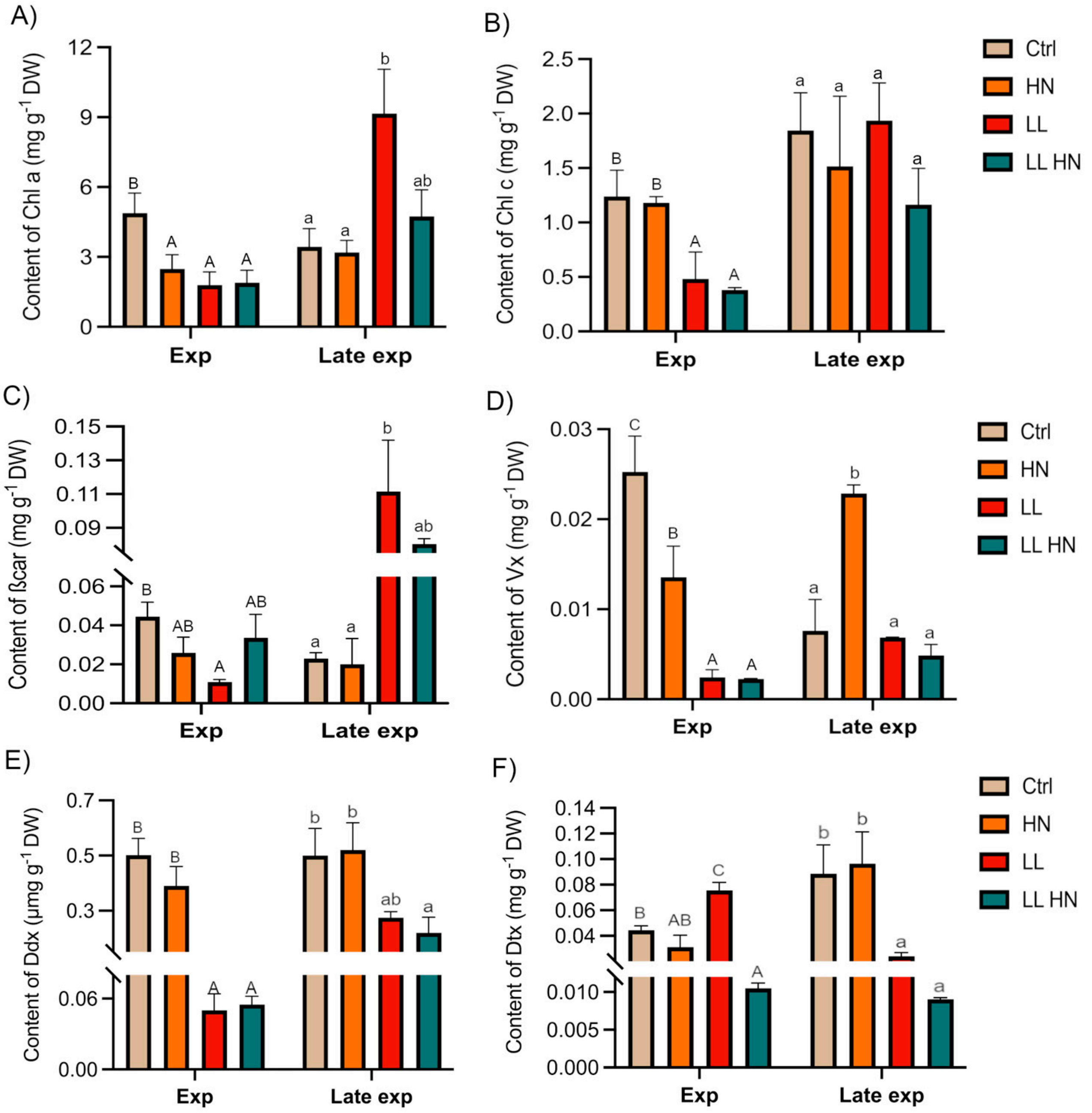

Figure S2: The three isoforms of predicted *Violaxanthin de-epoxidase like* were identified using Unipro UGENE 51.0 software and the NCBI BLAST database tool, were analysed with the most similar *VDL* of different diatoms using for the alignments using the MUSCLE algorithm inbuilt in Unipro UGENE 51.0. The aligned sequences were used to construct a Maximum likelihood tree with the Bootstrap method (1000 bootstrap) through MEGA11 (Molecular Evolutionary Genetics Analysis). The predicted VDLs of *T. rotula* were highlighted with a red circle.

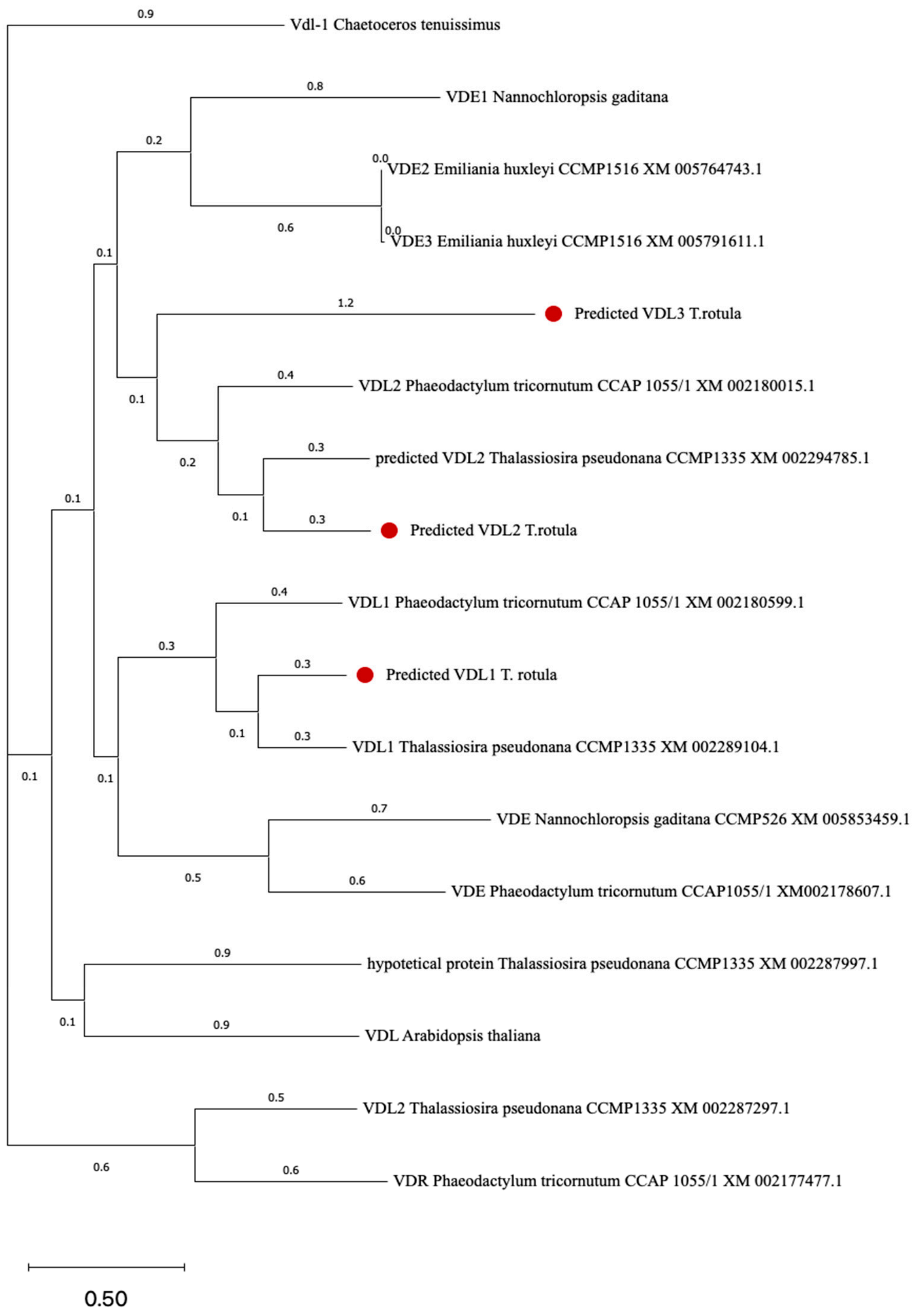

Supplement: Supplementary file 1 [file plants-14-03344-s001.zip › Figure Supplementary.pdf]
