# Supplementary material for: Influence of Nitrate and Light on Fucoxanthin Content and Key Gene Expression in the Marine Diatom Thalassiosira rotula
Source: Plants (Basel). 2025 Oct 31;14(21):3344. doi: 10.3390/plants14213344 (PMC12610687; doi:10.3390/plants14213344)
Supplement: Supplementary file 1 [file plants-14-03344-s001.zip › Table Supplementary.pdf]

Table S1: Primers used in the present study

List of primers tested	SEQUENCE 5′-3′ Forward	SEQUENCE 5′-3′ Reverse	Amplification
Phytoene Synthase (PSY1)	ATTGTAAGGTGAAGTATCCCGC	GGTCCCAGCCACACGATAAC	166 bp
Phytoene Synthase (PSY2)	TGAAGAGCAGCCAATCGGAC	ACATCATCCGTTATTTCGTCCA	185 bp
Phytoene Desaturase (PDS1)	GTGTGTTCCAGTCCCGTGATG	CTGGGTTGGTATGAGGCGTTTG	150 bp
Phytoene Desaturase (PDS2)	GATGCTTACGCTGGGTGAGAAA	CACCTCCTCGTTGATTCTCTCG	151 bp
Zeta Carotene Desaturase (ZDS)	GGCGTGACCGTGAATCTCTC	GGCAGCACCTTCTTGATTCCG	170 bp
Zeta Carotene Isomerase (ZCIS)	CCGTTCCAGGCTATTTGAGGC	CCACCAGCATTCAACCCCGA	166 bp
Carotenoid isomerase (CRTISO1)	GAGCGGCAAGGCAGTGGGA	GCCTTGACCCCGAGATGCA	237 bp
Carotenoid isomerase (CRTISO 2)	GCTCGCCTTTGACCCACGC	CTCCACTCAACGCCACAGCA	165 bp
Zeaxanthin Epoxidase (ZEP1)	CCCGTAGCATTGTGTGGCGA	CGAATGACACGAACACGGGAA	170 bp
Zeaxanthin Epoxidase (ZEP2)	CGTGAAGCGGCGGGACCTAT	GAACTCCCTCCAACTCTCGTC	170 bp
Violaxanthin De-Epoxidase like (VDL1)	GGCTAAGGCAGGGTTGGACT	ATACTCGCCACGCCATCCTG	181 bp
Violaxanthin De-Epoxidase like (VDL2)	GCGGACTGGTGGATGAAGCG	TCCCCTCCCAGCGATTCCC	155 bp
Violaxanthin De-Epoxidase like (VDL3)	CAATGGTGGTGTTGGGGAGAA	CTTGTCCTGGGTCATTCTCGC	205 bp
Diadinoxanthin De-Epoxidase (DDE)	GATACGGAGGTGCGGTGGTC	AATCGCCATCTTCCCCGCAAA	194 bp
Tubulina alfa (TUBa)	GTATCGCCAGCTCTACCATCC	GTGGCGTGGAAGATGAGGAATC	176 bp
Glyceraldehyde-3-phosphate dehydrogenase (GAPDH)	CTGCGAAGGTCCATCCACCGTC	CCACAAGGAGTACGAGAACAGC	172 bp
